# Exposure Profile and Characteristics of Parabens and Alkylphenols in Plasma among Rural Adults in Central China

**DOI:** 10.3390/toxics11110926

**Published:** 2023-11-13

**Authors:** Qian Gao, Changsheng Huan, Yu Song, Zexin Jia, Qingqing Cao, Chongjian Wang, Zhenxing Mao, Wenqian Huo

**Affiliations:** 1 Department of Occupational and Environmental Health, College of Public Health, Zhengzhou University, Zhengzhou 450001, China13849295236@163.com (Z.J.);; 2 Department of Epidemiology and Biostatistics, College of Public Health, Zhengzhou University, Zhengzhou 450001, China; tjwcj2005@126.com (C.W.);

**Keywords:** parabens, alkylphenols, exposure level, health risk assessment

## Abstract

Parabens and alkylphenols pose serious hazards to human health, yet there are few studies on their exposure profiles and health risks in rural Chinese populations. In this study, 804 participants were selected from the Henan Rural Cohort in mid-eastern China. The plasma levels of parabens (methylparaben, ethylparaben, propylparaben, butylparaben (BuP)) and alkylphenols (4-tert-butylphenol (4-t-BP), 4-tert-octylphenol (4-t-OP)) were analyzed via liquid chromatography–tandem mass spectrometry. Linear regression models were used to investigate factors that may influence pollutant exposure levels. The correlation between contaminants was assessed using Spearman’s correlation. The human contaminant intake was estimated using the estimated daily intake (EDI). The health risk was assessed using the hazard quotient (HQ). The detection frequency of four parabens and two alkylphenols exceeded 75%, with median concentrations of 0.444, 0.067, 0.078, 0.053, 8.810, and 6.401 ng/mL, respectively. Significant correlations were observed between parabens, as well as between 4-t-BP and 4-t-OP. Regarding gender, paraben concentrations were higher in women than in men, except for BuP. The *EDI* for pollutants except 4-t-OP was lower than their respective tolerable/acceptable daily intake. In total, 85.70% of participants had 4-t-OP HQ > 1. A widespread exposure to parabens and alkylphenols among the rural population was found. The high health risks of alkylphenol exposure indicate that alkylphenols should be used with caution.

## 1. Introduction

With rapid economic development and an acceleration in product iterations, contamination from environmental endocrine disruptors is becoming more widespread. Parabens and alkylphenols are common types of environmental endocrine disruptors and are widely used in everyday products. Parabens mainly include methylparaben (MeP), ethylparaben (EtP), propylparaben (PrP), butylparaben (BuP), and benzylparaben (BzP), which are often used as antimicrobial preservatives in food, pharmaceuticals, cosmetics, and personal care products (PCPs) [1]. The common alkylphenol derivatives 4-tert-butylphenol (4-t-BP) and 4-tert-octylphenol (4-t-OP) are ingredients in non-ionic surfactants, lubricants, and foaming agents and are, therefore, widely used in the production of soaps, cosmetics, paints, polycarbonate (PC), and insecticides [2,3].

Humans are exposed to environmental endocrine disruptors in many ways. Parabens enter the body mainly through ingestion, inhalation, and dermal absorption, whereas exposure to alkylphenols usually occurs through drinking water and food [1,4]. Because they readily enter the external environment, such as soil and water, through human activities, humans can also be exposed to them indirectly through secondary exposure [5]. Parabens and alkylphenols are usually metabolized in the liver and excreted in the urine after entering the body [6,7], so studies often use concentrations in urine or blood to understand recent internal human exposure to environmental contaminants. Urine, blood, and breast milk are considered good biological samples for assessing phenol exposure in humans [8]. However, the use of urine as a biological sample to assess phenol exposure usually requires adjustment for urinary creatinine, whereas the use of blood samples tends to be unaffected by creatinine and to not require correction. Although phenols tend to have a high detection rate in urine, an in vitro assay showed that straight-chain parabens are stable in plasma and that most parabens have a high level of plasma protein binding [9]. Free parabens remaining after initial metabolism can bind to human serum albumin to help them avoid being hydrolyzed and, thus, enter other tissues and accumulate in the organism [10]. This suggests that plasma concentrations in phenols are a stable reflection of recent exposure in organisms. In addition, for future comparison with exposure in urine, the detection of phenolic compounds in different biological samples was analyzed to identify more accurate samples for phenol biomonitoring. So, it is important to monitor the plasma concentrations of parabens and alkylphenols.

There is growing concern about environmental endocrine disruptors because of their potential hazards. Although parabens have a long history of use and are generally considered safe and low in toxicity, their effects on the health of organisms are gradually being reported [11]. Several recent in vivo studies have shown that parabens have the ability to accelerate ovarian aging, impede embryo implantation in mice, and interfere with epigenetic regulation in humans and rodents, and may cause reproductive toxicity and genotoxicity [12,13,14,15]. Further epidemiological investigations have found that parabens have weak estrogenic activity and can disrupt sex hormone levels in the body [16], even affecting prenatal fetal and child development and increasing the risk of breast cancer in women [17,18,19]. In addition, parabens have been found to disrupt the balance of thyroid hormones [20]. As for another common type of EDC, alkylphenols, several toxicological studies have shown that 4-t-BP and 4-t-OP can cause endocrine disruption and reproductive and developmental toxicity, and induce DNA damage and apoptosis in animals [3,21,22,23,24]. Epidemiological studies have shown that exposure to 4-t-OP may increase the likelihood of male infertility [25]. In addition, an investigation of occupational vitiligo patients found that 4-t-BP has a hypopigmentation effect [26].

Domestic and international studies have shown that paraben exposure levels vary from region to region [27]. In a study that reported paraben exposure levels in nine countries, the highest MeP, EtP PrP, and ToTPA (the sum of all parabens) concentrations in urine samples were found in South Korea, followed by China [28]. Additionally, 4-t-BP and 4-t-OP have been detected in large quantities in rivers and in industrial and domestic wastewater, which may pose a risk to human health [3]. However, there are limited studies related to their exposure for rural populations in China. Moreover, with the accelerated urbanization in China, people in rural areas may be increasingly exposed to pollutants [29]. Considering this along with the limited medical resources and poor awareness of protection in rural populations, it is necessary to obtain information on exposure to parabens and alkylphenols in rural Chinese populations to better prevent the health damage caused by paraben and alkylphenol exposure. 

China has a relatively large rural population [29], and there are fewer data on the exposure of rural populations to environmental pollutants, especially in central China. Therefore, in order to address the gap in this area, we organized this survey. Taking advantage of the Henan Rural Cohort Study, this study aimed to investigate the exposure profile of parabens and alkylphenols and assess their health risks based on plasma levels in a rural Chinese population. As a secondary objective, the factors that may influence participants’ exposure to contaminants were initially explored.

## 2. Methods

### 2.1. Study Population

The subjects of this study were recruited from the Henan Rural Cohort (registration number: ChiCTR-OOC-15006699), which has been described in detail in previous articles, including details on the selection of study subjects [30]. A total of 838 participants without impaired fasting glucose or diabetes, who took part in the baseline survey, were randomly selected. After removing outliers for contaminants (n = 31) and those missing values for body mass index (BMI) (n = 3), a total of 804 subjects were finally included in the present study.

Demographic characteristics, collected by trained investigators through standardized questionnaires, included age, gender, education level, average monthly income, marital status, smoking and alcohol status, and physical activity. BMI was calculated from participants’ height and weight, and it was equal to weight (kg)/height^2^ (m^2^). The age was divided into 18~45 years old, 46~65 years old, and >65 years old. Education level was divided into participants who had never attended school, those who attended primary school, those who attended junior secondary, and those who had a higher level of education than these. The average monthly income was divided into CNY <500, CNY 500~999, and CNY >1000. Marital status was divided into marriage/cohabiting and widowed/single/divorced. Smoking and alcohol status were each divided into current and never/past groups. Physical activity was divided into three groups: low, moderate, and high. BMI was classified as <18.5 kg/m^2^,18.5~23.9 kg/m^2^, and ≥24.0 kg/m^2^.

The research protocol was approved by the Life Sciences Ethics Committee of Zhengzhou University (code: [2015] MEC(S128)) and adhered to the principles of the Declaration of Helsinki.

### 2.2. Chemicals Analysis

Samples were collected in 2016, and venous blood was collected in the morning by uniformly trained medical personnel after participants had fasted for more than 8 h. Plasma samples were obtained via centrifugation at the survey site and stored in an ultra-low temperature refrigerator at −80 °C for testing. The sample pretreatment of parabens and alkylphenols was performed via liquid–liquid extraction. Simply, 200 μL L of plasma was mixed with 20 μL L of mixed internal standard, 200 μL of ultrapure water, 1 mL of acetonitrile, and 3 mL of hexane in a 15 mL Eppendorf (EP) tube. Then, the mixed solution was centrifuged at a low temperature (4 °C, 5515 rpm) for 10 min, and the supernatant was moved into a new EP tube. Subsequently, 1 mL of acetonitrile and 3 mL of a mixture of dichloromethane and hexane (1:1 volume) were added to the precipitate, mixed well via shaking, and centrifuged at a low temperature for 10 min. The supernatant was removed, and the procedure was repeated twice. The combined supernatant was blown using a nitrogen-blowing instrument until it was nearly dry. Finally, it was re-solubilized with 200 μL acetonitrile, vortexed, filtered through a 0.22 μm membrane into the injection vial, and stored at −20 °C for measurement.

The target compounds were analyzed via liquid chromatography–tandem mass spectrometry (LC-MS/MS) (Waters XEVO TQ-S system (Waters, Milford, MA, USA)). The separation was performed using an ACQUITY UPLC^®^ BEH C18 column (1.7 μm, 2.1 mm × 100 mm, Waters). The mobile phase consisted of pure water and methanol (mobile phase A: pure water; mobile phase B: methanol). The chromatographic column temperature was set at 40 °C. The chromatographic elution program was as follows: 0–0.5 min: 65% A and 35% B; 0.5–1.0 min: 65–40% A and 35–60% B, hold for 2 min; 3–5 min: 40–0% A and 60–100% B, hold for 3 min; 8–8.1 min: 0–65% A and 100–35% B, hold for 1.9 min. The total elution time was 10 min, the injection volume was 5 μL, and the liquid phase flow rate was 0.3 mL/min. The plasma paraben and alkylphenol levels were detected via mass spectrometry in negative ion electrospray mode and multiphase reaction detection mode. Additional details about the instrument conditions are available in Table S1.

The limits of detection (LOD) for each contaminant were MeP (0.0458 ng/mL), EtP (0.0001 ng/mL), PrP (0.0254 ng/mL), BuP (0.0156 ng/mL), BzP (0.0128 ng/mL), 4-t-BP (0.006 ng/mL), and 4-t-OP (0.1477 ng/mL).

### 2.3. Quality Control and Quality Assurance

In this study, three different levels of spiked samples were prepared, and six spiked samples were measured in parallel within a day to calculate the intra-day coefficient of variation. The spiked samples were measured once a day for three consecutive days to calculate the inter-day coefficient of variation. In addition, one solvent blank and one spiked sample per 12 samples were set up for quality control. The quantitative analysis was performed using an 8-point calibration curve with regression coefficients greater than 0.99 over the range of 0.01~50 ng/mL. In this experiment, the limit of detection (LOD) is defined as 3-fold the signal-to-noise ratio The limit of quantitation (LOQ) is defined as the 10-fold signal-to-noise ratio. The LOD for MeP, EtP, PrP, BuP, BzP, 4-t-BP, and 4-t-OP were 0.05, 0.0001, 0.03, 0.02, 0.01, 0.003, and 0.074 ng/mL, respectively. More detailed information, such as the spiked recoveries, can be found in Table S2.

### 2.4. Estimated Daily Intake and Health Risk Assessment

*EDI* (estimated daily intake) can be estimated from the concentration of contaminants in plasma through the following equation [31]:

(1) $$\mathrm{EDI}=\frac{C_{b}*V}{BW}\;(\mathrm{ng}/\mathrm{kg}\;\mathrm{bw}/\mathrm{day})$$

where *C_b_* is the individual concentration of parabens and alkyl phenols in the plasma (ng/mL), *V* is the average human plasma volume (about 1980 mL for adults) [32], and *BW* is the body weight (kg) of each participant.

The health risk assessment was based on each contaminant’s acceptable daily intake (ADI) or tolerable daily intake (TDI). Based on the European Food Safety Authority (EFSA, 2004) standards [28,33], the ADI for the sum of MeP and EtP (MeP+EtP) is 10,000,000 ng/kg bw/day, and the ADI for PrP is 100,000 ng/kg bw/day; the TDI for 4-t-OP according to the European Food Safety Authority (EFSA, 2015) is 0.067 ng/kg bw/day for men and 33.3 ng/kg bw/day for women [34].

The hazard quotient (HQ) method recommended by the U.S. Environmental Protection Agency (EPA) was used to quantify the risk of exposure to parabens and alkylphenols [35]:

(2) $$\mathrm{HQ}=\;\frac{EDI}{RfD}$$

Here, the *RfD* used the ADI or TDI values of parabens and alkylphenols. HQ > 1 indicates a potential exposure risk, and HQ ≤ 1 indicates no hazard.

To date, ADI or TDI values for BuP and 4-t-BP have not been determined, so their HQ values cannot be calculated. However, related studies have shown that the no-observed-adverse-effect-level (*NOAEL*)-calculated exposure margin (MOE) of pollutants can be used to assess their exposure risk [34]:

(3) $$\mathrm{MOE}=\;\frac{EDI}{NOAEL}$$

According to other relevant studies, the *NOAEL* of BuP is 2,000,000 ng/kg bw/day [11]. However, the *NOAEL* for 4-t-BP has not been determined, and only a basic characterization and comparison of 4-t-BP concentrations can be made.

### 2.5. Statistical Analysis

Contaminant levels below the LOD or not detected were replaced by half of the LOD. Compounds with a detection frequency (DF) less than 50% were not subjected to subsequent statistical analysis.

The Kolmogorov–Smirnov test was used to test whether the distribution of all variables in the study conformed to normality. Since parabens and alkylphenols had skewed distributions, they were transformed into the natural logarithm (Ln transformation). Normally distributed continuous variables were expressed as means (standard deviation, SD), non-normally distributed continuous variables were expressed as medians (interquartile range, IQR), and categorical variables were expressed as numbers (percentages). The Chi-square test and *t*-test were used to compare the differences in the distribution of demographic characteristics between men and women for categorical variables and continuous variables that conformed to a normal distribution, respectively. Mann–Whitney U tests were used for continuous variables that did not conform to a normal distribution. Spearman’s correlation coefficient was applied to test the correlation between the pollutants. Simple linear regression was used to estimate the relationship between pollutant concentrations and demographic characteristics and to compare the differences after stratification by gender.

Statistical analysis was performed using SPSS version 21.0 and R version 4.2. Two-sided test, *p*-value < 0.05 was considered statistically significant.

## 3. Results

### 3.1. Demographic Characteristics

The demographic characteristics of the participants are shown in Table 1. Among the 804 participants, there were 518 women (64.43%) and 286 men (35.57%). All were >18 years of age, with 46, 541, and 217 participants in each of the three age groups. In the total population, about 43.03% had received junior high school education or higher, 29.23% had an average monthly income of more than CNY 1000, and 88.31% had married or cohabiting status. Only a small number of people were current smokers (17.16%) and drinkers of alcohol (12.56%). Most people performed moderate physical activity every day (51.37%). The mean BMI of the participants was 23.53 kg/m^2^, and the mean BMI for men and women was 23.14 kg/m^2^ and 23.75 kg/m^2^, respectively. The differences in the distribution of education level, smoking, alcohol status, physical activity, and BMI between men and women were statistically significant (*p* < 0.05). Compared to the women, the men were more educated, and more likely to be smokers and drinkers, but had a lower BMI.

### 3.2. Plasma Concentrations, and Correlations of Parabens and Alkylphenols

Table 2 shows that in this study, except for BzP, the DFs of the other six pollutants (MeP, EtP, PrP, BuP, 4-t-BP, 4-t-OP) were all higher than 75%, so BzP was excluded from subsequent studies. The DF of 4-t-OP was the highest, reaching 100%. The median concentration of MeP (0.444 ng/mL) was the highest among the parabens, followed by PrP (0.078 ng/mL), EtP(0.067 ng/mL), and BuP(0.053 ng/mL), respectively. The median concentrations of 4-t-BP and 4-t-OP were 8.810 ng/mL and 6.401 ng/mL, respectively.

**Figure 1:**
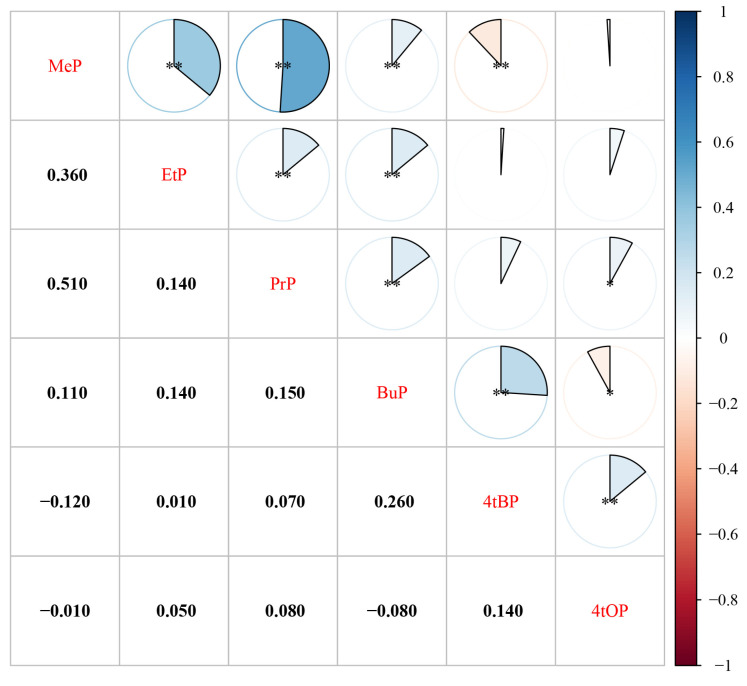
Spearman correlation coefficients among six pollutants. Abbreviation: MeP, methylparaben; EtP, ethylparaben; PrP, propylparaben; BuP, butylparaben; 4-t-BP, 4-tert-butylphenol; 4-t-OP, 4-t-octylphenol. Notes: * represents *p* < 0.05; ** represents *p* < 0.01.

Figure 1 shows the correlations between the target pollutants. Parabens showed a relatively strong correlation between MeP and PrP (r = 0.510, *p* < 0.01), and MeP was weakly correlated with EtP and BuP (r = 0.360, r = 0.110, respectively). EtP, PrP, and BuP were also weakly correlated with each other. There was a weak positive correlation between 4-t-BP and 4-t-OP (r = 0.140, *p* < 0.01). Between parabens and alkylphenols, 4-t-BP showed a significant negative correlation with MeP, and a significant positive correlation with BuP; however, 4-t-OP and parabens showed weak correlations.

### 3.3. Correlation between Demographic Characteristics and Pollutant Concentration

#### 3.3.1. Total Population

**Figure 2:**
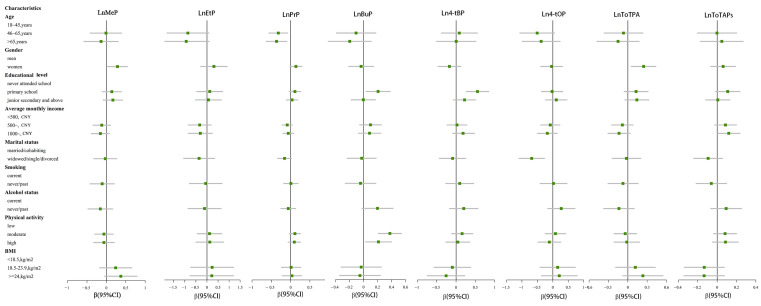
Forest plot of the correlations between the pollutant plasma concentrations and demographic characteristics.

The results of Figure 2 demonstrate that PrP concentrations in plasma decreased with increasing age, and MeP and ToTPA were higher in the plasma of women. Compared to those who had never attended school, BuP and 4-t-BP were higher in the population that had attended primary school. ToTAPs (β: 0.120, 95% CI: 0.004, 0.235; Table S3) were higher in the plasma of those with higher average monthly income levels (income > CNY 1000). The plasma concentrations of 4-t-OP (β: −0.684, 95% CI: −1.094, −0.274) were higher in people who were married and cohabitating. BuP concentrations increased with increased physical activity (*p* < 0.05). However, no significant relationships between smoking, alcohol status, BMI, and the studied pollutants were observed in the general population.

#### 3.3.2. Stratification by Gender

Further gender-specific analysis revealed that the concentration of 4-t-BP decreased with increasing BMI levels in men, but no such change was observed in women. After stratified analysis, the relationship between BuP and high physical activity lost its significance in both men and women. However, other pollutants related to the demographic characteristics of the total population remained relevant for women after stratification. In addition, alcohol status, which was not significant in the total population, showed a significant negative correlation with MeP (β: −0.963, 95% CI: −1.726, −0.200) and ToTPA (β: −0.671, 95% CI: −1.238, −0.104) in women who were never/past drinkers compared with current drinkers. The results of the gender-specific analyses are shown in Table S4.

### 3.4. Health Risk Assessment

The EDIs of each pollutant are shown in Table 3; 4-t-BP had the highest *EDI* (median: 295.366 ng/kg bw/day), followed by 4-t-OP (median: 138.775 ng/kg bw/day) and MeP (14.816 ng/kg bw/day). EtP, PrP, and BuP had the lower EDIs of 2.166 ng/kg bw/day, 2.643 ng/kg bw/day, and 1.761 ng/kg bw/day, respectively. After gender separation, 4-t-BP was still the highest *EDI* among men and women. In addition, there were significant differences in contaminant concentrations between men and women for all analyses. The maximum *EDI* values for MeP, EtP, and PrP did not exceed their limits (lower than the ADI for MeP+EtP: 10,000,000 ng/kg bw/day, and the ADI for PrP: 100,000 ng/kg bw/day). The results for BuP were similar to those described above, with the maximum value not exceeding its *NOAEL* (2,000,000 ng/kg bw/day). In contrast, the maximum values of *EDI* of 4-t-OP for both men and women were greater than their respective TDIs. Since there was no ADI, TDI, or *NOAEL* standard for 4-t-BP, no comparison could be made.

After calculation, it was found that no parabens had an HQ exceeding 1, but 689 participants with 4-t-OP had an HQ > 1, including 286 men (41.51%) and 403 women (58.49%).

## 4. Discussion

In this study, we investigated the exposure profiles of parabens and alkylphenols, their plasma concentration distribution in relation to demographic characteristics, and health risk assessments among a general rural population in Henan Province, China. The results revealed that parabens and alkylphenols were detected with high detection rates, except for BzP. The major contaminant species for parabens were MeP and PrP; however, the HQs for all parabens were less than 1, indicating a low health risk. The median concentrations for 4-t-BP (8.810 ng/mL) and 4-t-OP (6.401 ng/mL) were relatively high. In addition, 85.70% of participants had a 4-t-OP HQ > 1, indicating that special attention should be paid to exposure to alkylphenols in daily lives Correlation analyses with demographic characteristics revealed that parabens and alkylphenols were related to several factors, such as gender, age, education level, average monthly income, and physical activity.

The DFs of MeP and PrP were high in this investigation, with the highest concentration of MeP in plasma samples, followed by PrP, EtP, and BuP, respectively, which is consistent with most studies [11,36,37,38]. Although the concentration of MeP (median: 0.444 ng/mL) was 5–6 times higher than those of other parabens, it was also much lower than the concentration of MeP in plasma in Malaysians (mean: 7.7 ng/mL) and in plasma in pregnant Indian women (median: 13.18 ng/mL) [6,39]. The reason for this may be the low frequency of use of foods and personal care products containing parabens, etc., in rural Chinese populations [29,40,41,42]. The DF of EtP in this study was approximately 80%, which is comparatively lower than that reported in studies conducted in Wuhan, Jingyuan County, Wuxi, and Taishun (where the DF of EtP was close to 100%) [29,38,43]. This discrepancy may be attributed to the relatively low usage of PCPs such as creams, body washes, and hand soaps among rural populations [29,38,43]. Compared with studies conducted in foreign countries, our results were roughly similar to those from the United States, Denmark, and Germany [11,37]. However, compared with studies in Japan and Korea, where EtP was detected at higher concentrations, the proportions of parabens in the urine of Japanese and Korean people were in the following order: MeP> EtP> PrP> BuP. The proportions of parabens in our resultant plasma were, in order, MeP > PrP > EtP > BuP. The reason for this difference may be related to the different composition of parabens in PCPs or different washing habits in China, Korea, and Japan, or the Korean preference for saltier foods such as kimchi [44,45,46]. Linear regression results showed a significant negative correlation between PrP and age, and analysis stratified by sex showed that the difference between PrP and age was mainly found in women. In addition, MeP concentrations were significantly higher in women than in men. It has been previously reported that women use PCPs more frequently than men, and that MeP and PrP are also predominantly present in PCPs, such as cosmetics and lotions [27,45].

To our knowledge, this is the first study to investigate the exposure levels of 4-t-BP and 4-t-OP simultaneously. The median concentration of 4-t-OP in our study was 6.401 ng/mL, which was approximately seven times lower than control plasma concentrations in a Korean case–control study (mean: 39.37 ng/mL) and maternal serum concentrations in the Guangxi Zhuang birth cohort (median: 45.91 ng/mL) [47,48]. The reasons for this difference may be: (1) the lower LOD of 4-t-OP in this study; (2) the food in the studied region possibly being less likely to be contaminated with alkylphenols [47].

In our study, the HQ values of parabens were <1, but 85.70% of participants had an HQ > 1 for 4-t-OP. These results are consistent with the findings from Denmark (the *EDI* of all parabens was less than the TDI) and Turkey (HQ for 4-t-OP: 2.000 for men and 5.306 for women) [34,37]. However, the high HQ of 4-t-OP may be due to the fact that there are fewer studies on 4-t-OP exposure limits, and the existing data on exposure limits are not widely accepted internationally (unlike those for parabens), so the assessed exposure levels may be less accurate [34,37]. Due to the lack of population-based epidemiological investigation into 4-t-BP, it was not possible to compare the exposure level and health risk. However, via analogy with the results of 4-t-OP, it can be assumed that the exposure risk of 4-t-BP was also high in the rural population in Henan Province. The exposure limits of parabens and alkylphenols need to be further investigated. The high HQ for 4-t-OP suggests that rural populations should be careful to avoid exposure to and use of 4-t-OP in their daily life. Although none of the HQs for parabens exceeded 1, this does not mean that we can ignore their exposure, considering the hazards they pose to humans. The findings of this study contribute to the study of the exposure to, and associations of, parabens in rural areas of China, but more detailed exposure and mechanism studies need to be conducted.

## 5. Conclusions

Through surveying the general population in rural China, we found that the population was widely exposed to parabens and alkylphenols. The plasma paraben levels were low in the population, but the levels of alkylphenols were high. In addition, we found that plasma concentrations were associated with factors such as gender, education level, and physical activity. In the present study, the EDIs of parabens were all lower than their TDI/ADI. However, alkylphenols were associated with relatively high health risks, with 85.7% of the participants having an HQ of 4-t-OP > 1. Therefore, further studies are needed to prioritize risk management measures for 4-t-BP and 4-t-OP. However, the risk of parabens cannot be completely ignored, and there is a need to continue to study the possible health hazards of parabens and alkylphenols and the possible factors affecting their levels. Our findings may provide a scientific and theoretical basis for future interventions into paraben and alkylphenol exposure and the development of health risk thresholds for related pollutants.

## Figures and Tables

**Table 1:** Demographic characteristics of general adults from rural areas in China.

Characteristics	Whole	Men	Women	*p*-Value ^a^
	n = 804	n = 286	n = 518	
**Age, years, median (IQR)**	61 (54,66)	61 (54,66)	61 (54,66)	0.935
18~45	46 (5.72)	13 (4.55)	33 (6.37)	
46~65	541 (67.29)	197 (68.88)	344 (66.41)	
>65	217 (26.99)	76 (26.57)	141 (27.22)	
**Education level, n (%)**				<0.001
never attended school	211 (26.24)	45 (15.73)	166 (32.05)	
primary school	247 (30.72)	74 (25.87)	173 (33.40)	
junior secondary and above	346 (43.03)	167 (58.39)	179 (34.55)	
**Average monthly income, n (%)**				0.553
CNY <500	311 (38.68)	104 (36.36)	207 (39.96)	
CNY 500~	258 (32.09)	93 (32.52)	165 (31.85)	
CNY 1000~	235 (29.23)	89 (31.12)	146 (28.19)	
**Marital status, n (%)**				0.920
married/cohabiting	710 (88.31)	253 (88.46)	457 (88.22)	
widowed/single/divorced	94 (11.69)	33 (11.54)	61 (11.78)	
**Smoking, n (%)**				<0.001
current	138 (17.16)	135 (47.20)	3 (0.58)	
never/past	666 (82.84)	151 (52.80)	515(99.42)	
**Alcohol status, n (%)**				<0.001
current	101 (12.56)	89 (31.12)	12(2.32)	
never/past	703 (87.44)	197 (68.88)	506(97.68)	
**Physical activity, n (%)**				<0.001
low	188 (23.38)	92 (32.17)	96 (18.53)	
moderate	413 (51.37)	108 (37.76)	305 (58.88)	
high	2043 (25.25)	87 (30.07)	117 (22.59)	
**BMI, kg/m^2^, mean ± SD**	23.53 ± 3.30	23.14 ± 3.05	23.75 ± 3.41	0.014
<18.5	45 (5.60)	16 (5.59)	29 (5.60)	
18.5–23.9	412 (51.24)	162 (56.64)	250 (48.26)	
≥24	347 (43.16)	108 (37.76)	239 (46.14)	

Abbreviation: SD, standard deviation; IQR, interquartile range; BMI, body mass index; CNY, Chinese yuan. ^a^ Differences in continuous and categorical covariates conforming to normal distribution between men and women were tested via *t*-test and Chi-square test, respectively. Continuous variables not conforming to normal distribution were tested using Mann–Whitney U tests.

**Table 2:** Plasma concentrations (ng/mL) of parabens and alkylphenols in participants.

Pollutants	Group	DF (%)	25th	50th	75th	Max	LOD
MeP	whole	92.91	0.185	0.444	1.008	28.842	0.0458
	men	92.66	0.160	0.352	0.880	28.842	
	women	93.05	0.193	0.488	1.087	28.787	
EtP	whole	78.98	0.018	0.067	0.125	9.136	0.0001
	men	77.62	0.015	0.058	0.099	9.136	
	women	79.73	0.023	0.074	0.131	4.630	
PrP	whole	89.18	0.057	0.078	0.107	3.112	0.0254
	men	87.76	0.055	0.074	0.100	1.171	
	women	89,96	0.059	0.080	0.110	3.112	
BuP	whole	79.48	0.026	0.053	0.074	1.269	0.0156
	men	77.27	0.019	0.052	0.071	0.804	
	women	80.69	0.027	0.055	0.077	1.269	
BzP	whole	2.99	<LOD	<LOD	<LOD	<LOD	0.0128
	men	2.80	<LOD	<LOD	<LOD	<LOD	
	women	3.09	<LOD	<LOD	<LOD	<LOD	
4-t-BP	whole	97.39	4.863	8.810	15.068	99.436	0.0063
	men	98.26	4.674	8.511	14.086	99.436	
	women	96.91	4.915	8.988	15.399	95.488	
4-t-OP	whole	100.00	2.914	6.401	8.664	134.368	0.1477
	men	100.00	2.534	5.442	7.386	134.368	
	women	100.00	3.139	6.957	9.466	115.956	
ToTPA ^b^	whole		0.002	0.004	0.008	0.240	
	men		0.002	0.004	0.007	0.210	
	women		0.002	0.005	0.009	0.240	
ToTAPs ^b^	whole		0.061	0.091	0.154	0.800	
	men		0.057	0.089	0.140	0.800	
	women		0.062	0.095	0.161	0.730	

Abbreviation: DF, detection frequency; LOD, limits of detection; MeP, methylparaben; EtP, ethylparaben; PrP, propylparaben; BuP, butylparaben; BzP, benzylparaben; 4-t-BP, 4-tert-butylphenol; 4-t-OP, 4-t-octylphenol; ToTPA, the sum of all parabens; ToTAPs, the sum of alkylphenols. ^b^ indicates that the concentration of the pollutant is μmol/L.

**Table 3:** Estimated daily intake (*EDI*, ng/kg bw/day) and health risk assessment for exposure to parabens and alkylphenols among the study populations.

Pollutants	Whole				Men				Women				*p*-Value ^c^	N (%)
	25th	50th	75th	Max	25th	50th	75th	Max	25th	50th	75th	Max		HQ > 1
MeP	6.092	14.816	32.788	1071.437	4.989	11.787	27.918	1071.437	6.929	16.988	36.316	946.829	0.001	-
EtP	0.645	2.166	4.005	332.527	0.417	1.844	3.347	332.527	0.783	2.559	4.578	152.288	0.000	-
PrP	1.805	2.643	3.792	102.357	1.592	2.291	3.316	42.618	1.972	2.828	4.024	102.357	0.000	-
BuP	0.862	1.761	2.539	41.739	0.585	1.602	2.327	29.278	0.956	1.885	2.658	41.739	0.000	-
4-t-BP	163.121	295.366	512.503	3329.595	146.706	269.091	448.849	2773.016	178.007	311.857	544.479	3329.595	0.019	689 (85.70)
4-t-OP	42.973	138.775	299.862	4993.825	40.085	117.782	241.106	3924.032	46.111	151.107	350.961	4993.825	0.014	-

Abbreviations: MeP, methylparaben; EtP, ethylparaben; PrP, propylparaben; BuP, butylparaben; BzP, benzylparaben; 4-t-BP, 4-tert-butylphenol; 4-t-OP, 4-t-octylphenol. ^c^ Differences in pollutant levels between men and women were tested via simple linear regressions.

## Data Availability

The data that support the findings of this study are available upon reasonable request from the corresponding authors.

## Supplementary Materials

The following supporting information can be downloaded at: https://www.mdpi.com/article/10.3390/toxics11110926/s1, Table S1: HPLC-MS/MS optimized parameters for parabens and alkylphenols; Table S2: Spiked recoveries, coefficients of variation and detection limits of parabens and alkylphenols; Table S3: Association between plasma concentrations of targeted pollutants and demographic categories(n = 804); Table S4: Association between plasma concentrations of targeted pollutants and demographic categories by gender.
