# Supplementary material for: Exposure Profile and Characteristics of Parabens and Alkylphenols in Plasma among Rural Adults in Central China
Source: Toxics. 2023 Nov 13;11(11):926. doi: 10.3390/toxics11110926 (PMC10675222; doi:10.3390/toxics11110926)
Supplement: Supplementary file 1 [file toxics-11-00926-s001.zip › toxics-2675683-supplementary.pdf]

Supplementary Materials

Table S1 HPLC-MS/MS optimized parameters for parabens and alkylphenols.

Table S2 Spiked recoveries, coefficients of variation and detection limits of parabens and alkylphenols.

Table S3 Association between plasma concentrations of targeted pollutants and demographic categories(n = 804).

Table S4 Association between plasma concentrations of targeted pollutants and demographic categories by gender.

Table S1 HPLC-MS/MS optimized parameters for parabens and alkylphenols.

pollutants	Molecular weight	Retention time (min)	Parent ion (m/z)	Product ion (m/z)	Cone voltage (eV)	Collision energy (eV)
MeP	152	2.70	150.99	91.93	12	14
EtP	166	3.22	165.01	92.02	12	12
PrP	180	4.00	179.04	92.11	26	16
BuP	194	5.05	193.07	92.01	16	16
BzP	228	5.11	227.02	91.94	28	14
4-t-BP	150	5.20	149.07	133.31	2	18
4-t-OP	206	6.53	205.14	133.41	6	20

Table S2 Spiked recoveries, coefficients of variation and detection limits of parabens and alkylphenols.

pollutants	Recoveries (%)	Intra-day precision RSD (%)			Inter-day precision RSD (%)			LOD (ng/mL)
		$0.5^{a}/10^{b}$	$1.0^{a}/20^{b}$	$2.0^{a}/50^{b}$	$0.5^{a}/10^{b}$	$1.0^{a}/20^{b}$	$2.0^{a}/50^{b}$	
MeP	102.44	4.27	8.32	8.51	13.41	10.63	7.12	0.05
EtP	98.04	6.21	4.42	4.13	10.23	7.89	10.55	0.0001
PrP	97.74	4.20	4.21	4.62	1.62	7.71	7.34	0.03
BuP	109.50	9.09	5.51	7.54	7.80	8.69	6.45	0.02
BzP	109.56	6.21	8.67	6.05	39.62	25.51	12.64	0.01
4-t-BP	119.62	9.22	4.90	3.94	9.80	14.39	1.73	0.003
4-t-OP	117.86	5.01	6.04	2.41	7.89	8.34	1.47	0.074

a: the concentrations of parabens standard samples are 0.5 ng/mL, 1.0 ng/mL and 2.0 ng/mL, respectively.

b: the concentrations of alkylphenols standard samples are 10 ng/mL, 20 ng/mL and 50 ng/mL, respectively.

Table S3 Association between plasma concentrations of targeted pollutants and demographic categories(n=804).

Characteristics	β (95% CI)							
	LnMeP	LnEtP	LnPrP	LnBuP	LnToTPA	Ln4-t-BP	Ln4-t-OP	LnToTAPs
Age								
18~45, years	ref	ref	ref	ref	ref	ref	ref	ref
46~65, years	-0.011(-0.416,0.393)	-0.863(-1.811,0.085)	**-0.319(-0.561,-0.076)***	-0.106(-0.385,0.174)	-0.067(-0.366,0.233)	0.086(-0.380,0.552)	-0.506(-1.066,0.053)	-0.002(-0.204,0.201)
>65, years	-0.138(-0.579,0.302)	-0.938(-1.971,0.095)	**-0.364(-0.628,-0.100)****	-0.196(-0.500,0.109)	-0.151(-0.478,0.175)	0.004(-0.504,0.512)	-0.378(-0.987,0.232)	0.048(-0.172,0.268)
Gender								
men	ref	ref	ref	ref	ref	ref	ref	ref
women	**0.286(0.031,0.540)***	0.314(-0.283,0.911)	0.138(-0.015,0.291)	-0.034(-0.210,0.142)	**0.245(0.056,0.433)***	-0.172(-0.466,0.122)	-0.038(-0.391,0.314)	0.062(-0.066,0.189)
Educational attainment								
never attended school	ref	ref	ref	ref	ref	ref	ref	ref
primary school	0.139(-0.110,0.388)	0.124(-0.460,0.708)	0.112(-0.037,0.262)	**0.208(0.035,0.380)***	0.128(-0.057,0.312)	**0.555(0.268,0.843)****	-0.023(-0.368,0.321)	0.109(-0.015,0.234)
junior secondary and above	0.168(-0.082,0.418)	0.065(-0.521,0.651)	0.038(-0.112,0.188)	-0.002(-0.175,0.171)	0.140(-0.046,0.325)	0.220(-0.068,0.509)	0.112(-0.234,0.458)	0.007(-0.118,0.132)
Average monthly income								
<500, RMB	ref	ref	ref	ref	ref	ref	ref	ref
500~, RMB	-0.120(-0.343,0.103)	-0.330(-0.853,0.192)	-0.088(-0.221,0.046)	0.100(-0.054,0.254)	-0.085(-0.250,0.080)	0.024(-0.233,0.281)	-0.081(-0.389,0.227)	0.086(-0.025,0.198)
1000~, RMB	-0.155(-0.386,0.075)	-0.302(-0.842,0.238)	-0.060(-0.198,0.078)	0.086(-0.073,0.245)	-0.136(-0.307,0.034)	0.182(-0.084,0.477)	-0.177(-0.495,0.142)	**0.120(0.004,0.235)***
Marital status								
married/cohabiting	ref	ref	ref	ref	ref	ref	ref	ref
widowed/single/divorced/	-0.029(-0.325,0.268)	-0.357(-1.052,0.339)	-0.157(-0.335,0.020)	-0.026(-0.231,0.179)	-0.022(-0.242,0.198)	-0.089(-0.431,0.253)	**-0.684(-1.094,-0.274)****	-0.093(-0.241,0.055)
Smoking								
current	ref	ref	ref	ref	ref	ref	ref	ref
never/past	-0.109(-0.423,0.205)	-0.057(-0.794,0.679)	0.008(-0.181,0.196)	-0.042(-0.259,0.175)	-0.075(-0.307,0.158)	0.095(-0.267,0.457)	0.022(-0.413,0.456)	-0.060(-0.217,0.097)
Alcohol status								
current	ref	ref	ref	ref	ref	ref	ref	ref
never/past	-0.157(-0.476,0.161)	-0.109(-0.855,0.638)	-0.061(-0.253,0.130)	0.199(-0.021,0.420)	-0.138(-0.374,0.098)	0.200(-0.168,0.567)	0.274(-0.167,0.714)	0.093(-0.066,0.252)
Physical activity								
low	ref	ref	ref	ref	ref	ref	ref	ref
moderate	-0.062(-0.298,0.174)	0.116(-0.437,0.670)	0.111(-0.031,0.252)	**0.376(0.212,0.539)****	-0.040(-0.215,0.135)	0.157(-0.115,0.430)	0.086(-0.241,0.412)	0.082(-0.036,0.200)
high	-0.063(-0.329,0.203)	0.127(-0.498,0.751)	0.097(-0.063,0.256)	**0.213(0.029,0.397)***	-0.017(-0.214,0.180)	0.046(-0.261,0.354)	-0.112(-0.480,0.257)	0.086(-0.047,0.219)
BMI								
<18.5, kg/m2	ref	ref	ref	ref	ref	ref	ref	ref
18.5-23.9, kg/m2	0.238(-0.176,0.652)	0.235(-0.735,1.205)	0.013(-0.235,0.261)	-0.033(-0.319,0.253)	0.117(-0.189,0.424)	-0.096(-0.573,0.381)	0.156(-0.416,0.728)	-0.133(-0.339,0.074)
>=24.0, kg/m2	0.372(-0.047,0.791)	0.217(-0.765,1.199)	0.035(-0.217,0.286)	-0.051(-0.340,0.239)	0.233(-0.077,0.543)	-0.257(-0.740,0.226)	0.210(-0.369,0.789)	-0.134(-0.344,0.075)

"*" represents $P < 0.05$; "**" represents $P < 0.01$.

Table S4 Association between plasma concentrations of targeted pollutants and demographic categories by gender.

Characteristics	β (95% CI)							
	LnMeP	LnEtP	LnPrP	LnBuP	LnToTPA	Ln4-t-BP	Ln4-t-OP	LnToTAPs
**Men**								
Age								
18~45, years	ref	ref	ref	ref	ref	ref	ref	ref
46~65, years	0.491(-0.289,1.271)	-0.602(-2.420,1.217)	-0.226(-0.663,0.211)	-0.069(-0.618,0.480)	0.256(-0.319,0.831)	-0.352(-1.120,0.416)	-0.360(-1.389,0.669)	-0.062(-0.436,0.312)
>65, years	0.511(-0.328,1.351)	0.054(-1.902,2.010)	-0.253(-0.722,0.217)	-0.237(-0.828,0.353)	0.283(-0.336,0.901)	-0.558(-1.384,0.268)	-0.286(-1.393,0.821)	0.007(-0.395,0.410)
Educational attainment								
never attended school	ref	ref	ref	ref	ref	ref	ref	ref
primary school	-0.053(-0.578,0.473)	-0.139(-1.364,1.087)	0.067(-0.228,0.361)	0.034(-0.336,0.404)	-0.002(-0.389,0.385)	0.423(-0.094,0.941)	0.085(-0.608,0.779)	0.171(-0.081,0.423)
junior secondary and above	-0.004(-0.504,0.496)	-0.554(-1.720,0.612)	-0.023(-0.303,0.257)	-0.196(-0.548,0.156)	-0.029(-0.397,0.340)	0.009(-0.483,0.501)	-0.129(-0.789,0.531)	-0.026(-0.266,0.214)
Average monthly income								
<500, RMB	ref	ref	ref	ref	ref	ref	ref	ref
500~, RMB	-0.215(-0.606,0.177)	-0.202(-1.115,0.710)	-0.178(-0.397,0.042)	0.036(-0.240,0.311)	-0.154(-0.443,0.134)	**-0.478(-0.863,-0.092)***	-0.119(-0.635,0.398)	-0.093(-0.281,0.095)
1000~, RMB	-0.152(-0.553,0.250)	-0.280(-1.216,0.656)	-0.086(-0.311,0.139)	0.013(-0.269,0.296)	-0.128(-0.424,0.167)	-0.186(-0.581,0.209)	-0.347(-0.876,0.183)	-0.033(-0.225,0.160)
Marital status								
married/cohabiting	ref	ref	ref	ref	ref	ref	ref	ref
widowed/single/divorced/	-0.106(-0.620,0.407)	0.481(-0.717,1.678)	-0.094(-0.381,0.194)	0.098(-0.264,0.459)	-0.066(-0.445,0.313)	0.011(-0.494,0.517)	-0.188(-0.865,0.490)	0.006(-0.240,0.252)
Smoking								
current	ref	ref	ref	ref	ref	ref	ref	ref
never/past	-0.063(-0.396,0.271)	0.138(-0.640,0.916)	0.048(-0.139,0.235)	-0.032(-0.266,0.203)	-0.040(-0.286,0.206)	0.052(-0.276,0.380)	0.036(-0.405,0.476)	-0.102(-0.262,0.058)
Alcohol status								
current	ref	ref	ref	ref	ref	ref	ref	ref
never/past	-0.093(-0.458,0.273)	-0.547(1.399,0.306)	-0.106(-0.311,0.098)	0.147(-0.110,0.404)	-0.112(-0.382,0.157)	0.083(-0.277,0.442)	0.139(-0.344,0.621)	0.078(-0.098,0.253)
Physical activity								
low	ref	ref	ref	ref	ref	ref	ref	ref
moderate	0.226(-0.163,0.616)	0.622(-0.286,1.529)	0.179(-0.039,0.397)	**0.363(0.089,0.637)***	0.201(-0.086,0.488)	-0.106(-0.489,0.277)	-0.009(-0.522,0.505)	-0.008(-0.195,0.178)
high	0.019(-0.385,0.423)	0.179(-0.763,1.120)	0.127(-0.100,0.353)	0.192(-0.092,0.476)	0.042(-0.256,0.339)	-0.072(-0.469,0.326)	-0.384(-0.917,0.149)	-0.025(-0.218,0.169)
BMI								
<18.5, kg/m2	ref	ref	ref	ref	ref	ref	ref	ref
18.5-23.9, kg/m2	0.173(-0.544,0.890)	0.298(-1.373,1.968)	-0.087(-0.488,0.314)	-0.117(-0.621,0.388)	0.061(-0.467,0.589)	**-0.714(-1.420,-0.009)***	-0.102(-1.048,0.843)	-0.254(-0.598,0.089)
>=24, kg/m2	-0.033(-0.774,0.708)	0.223(-1.504,1.950)	-0.218(-0.633,0.196)	-0.198(-0.719,0.324)	-0.062(-0.607,0.484)	**-0.769(-1.497,-0.040)***	0.150(-0.827,1.127)	-0.121(-0.476,0.234)
**Women**								
Age								
18~45, years	ref	ref	ref	ref	ref	ref	ref	ref
46~65, years	-0.099(-0.571,0.373)	-0.818(-1.934,0.298)	**-0.315(-0.613,-0.017)***	-0.100(-0.429,0.229)	-0.106(-0.457,0.245)	0.269(-0.321,0.859)	-0.560(-1.239,0.119)	0.024(-0.220,0.268)
>65, years	-0.338(-0.854,0.178)	**-1.268(-2.486,-0.050)***	**-0.384(-0.709,-0.059)***	-0.160(-0.519,0.199)	-0.276(-0.659,0.107)	0.213(-0.431,0.858)	-0.397(-1.138,0.344)	0.060(-0.206,0.326)
Educational attainment								
never attended school	ref	ref	ref	ref	ref	ref	ref	ref
primary school	0.200(-0.082,0.483)	0.124(-0.544,0.792)	0.126(-0.052,0.304)	**0.268(0.071,0.465)****	0.166(-0.044,0.376)	**0.613(0.259,0.966)****	-0.101(-0.507,0.306)	0.086(-0.059,0.232)

junior secondary and above	0.291(-0.003,0.584)	0.374(-0.320,1.067)	0.080(-0.105,0.265)	0.068(-0.137,0.272)	**0.253(0.035,0.471)***	0.308(-0.059,0.674)	0.225(-0.197,0.647)	0.029(-0.122,0.181)
Average monthly income								
<500, RMB	ref	ref	ref	ref	ref	ref	ref	ref
500~, RMB	-0.025(-0.296,0.246)	-0.373(-1.013,0.267)	-0.026(-0.196,0.145)	0.128(-0.061,0.317)	-0.018(-0.219,0.183)	0.251(-0.087,0.589)	-0.110(-0.499,0.280)	**0.158(0.018,0.297)***
1000~, RMB	-0.163(-0.447,0.121)	-0.375(-1.046,0.296)	-0.051(-0.230,0.128)	0.100(-0.098,0.298)	-0.149(-0.360,0.062)	0.318(-0.037,0.673)	-0.166(-0.574,0.243)	**0.172(0.025,0.319)***
Marital status								
married/cohabiting	ref	ref	ref	ref	ref	ref	ref	ref
widowed/single/divorced/	0.031(-0.333,0.396)	-0.755(-1.616,0.106)	-0.190(-0.420,0.040)	-0.101(-0.355,0.153)	0.013(-0.258,0.284)	-0.098(-0.553,0.357)	**-0.933(-1.457,-0.409)****	-0.119(-0.307,0.069)
Smoking								
current	ref	ref	ref	ref	ref	ref	ref	ref
never/past	-0.352(-1.870,1.167)	-1.291(-4.878,2.297)	-0.278(-1.236,0.679)	0.521(-0.537,1.579)	-0.195(-1.323,0.933)	0.716(-1.181,2.613)	0.036(-2.147,2.219)	0.244(-0.540,1.028)
Alcohol status								
current	ref	ref	ref	ref	ref	ref	ref	ref
never/past	**-0.963(-1.726,-0.200)***	0.730(-1.073,2.533)	-0.020(-0.501,0.461)	0.311(-0.221,0.842)	**-0.671(-1.238,-0.104)***	0.532(-0.422,1.485)	0.456(-0.641,1.553)	-0.012(-0.406,0.382)
Physical activity								
low	ref	ref	ref	ref	ref	ref	ref	ref
moderate	-0.208(-0.511,0.096)	-0.181(-0.898,0.535)	0.076(-0.116,0.267)	**0.395(0.183,0.606)****	-0.165(-0.390,0.060)	0.302(-0.077,0.681)	0.151(-0.285,0.587)	0.128(-0.028,0.285)
high	-0.137(-0.495,0.221)	0.017(-0.829,0.863)	0.070(-0.156,0.296)	0.235(-0.014,0.485)	-0.076(-0.342,0.190)	0.135(-0.312,0.583)	0.053(-0.462,0.568)	0.153(-0.031,0.338)
BMI								
<18.5, kg/m2	ref	ref	ref	ref	ref	ref	ref	ref
18.5-23.9, kg/m2	0.301(-0.205,0.808)	0.271(-0.926,1.469)	0.059(-0.261,0.378)	0.014(-0.339,0.367)	0.173(-0.203,0.550)	0.208(-0.425,0.841)	0.318(-0.411,1.046)	-0.065(-0.327,0.197)
>=24, kg/m2	**0.612(0.104,1.121)***	0.339(-0.863,1.541)	0.165(-0.156,-.486)	0.036(-0.319,0.390)	**0.415(0.037,0.792)***	0.039(-0.596,0.674)	0.291(-0.440,1.022)	-0.117(-0.379,0.146)

"*" represents $P < 0.05$; "**" represents $P < 0.01$.
